# Nectin-like molecule-4/cell adhesion molecule 4 inhibits the ligand-induced dimerization of ErbB3 with ErbB2

**DOI:** 10.1038/s41598-017-10107-5

**Published:** 2017-09-12

**Authors:** Kiyohito Mizutani, Shin Kedashiro, Masahiro Maruoka, Yuki Ueda, Yoshimi Takai

**Affiliations:** 1 Division of Pathogenetic Signaling, Department of Biochemistry and Molecular Biology, Kobe University Graduate School of Medicine, 1-5-6 Minatojima-minamimachi, Chuo-ku, Kobe, Hyogo, 650-0047 Japan; 2 Health Metrics Development Team, RIKEN Compass to Healthy Life Research Complex Program, 6-7-1 Minatojima-minamimachi, Chuo-ku, Kobe, Hyogo, 650-0047 Japan; 3 Pathophysiological and Health Science Team, RIKEN Center for Life Science Technologies, 6-7-3 Minatojima-minamimachi, Chuo-ku, Kobe, Hyogo, 650-0047 Japan

## Abstract

The ligand-induced dimerization of cell surface single-transmembrane receptors is essential for their activation. However, physiological molecules that inhibit their dimerization and activation have not been identified. ErbB3 dimerizes with ErbB2 upon binding of heregulin (HRG) to ErbB3, causing the ErbB2-catalyzed tyrosine phosphorylation of ErbB3, which leads to the activation of the signalling pathways for cell movement and survival. Genetic disorders of this receptor cause tumorigenesis and metastasis of cancers. We show here that nectin-like molecule-4/cell adhesion molecule 4, known to serve as a tumour suppressor, interacts with ErbB3 in the absence of HRG and inhibits the HRG-induced dimerization of ErbB3 with ErbB2 and its activation. The third immunoglobulin-like domain of nectin-like molecule-4 *cis*-interacts with the extracellular domain 3 of ErbB3. We describe here a novel regulatory mechanism for the activation and signalling of cell surface single-transmembrane receptors.

## Introduction

**Figure 1:**
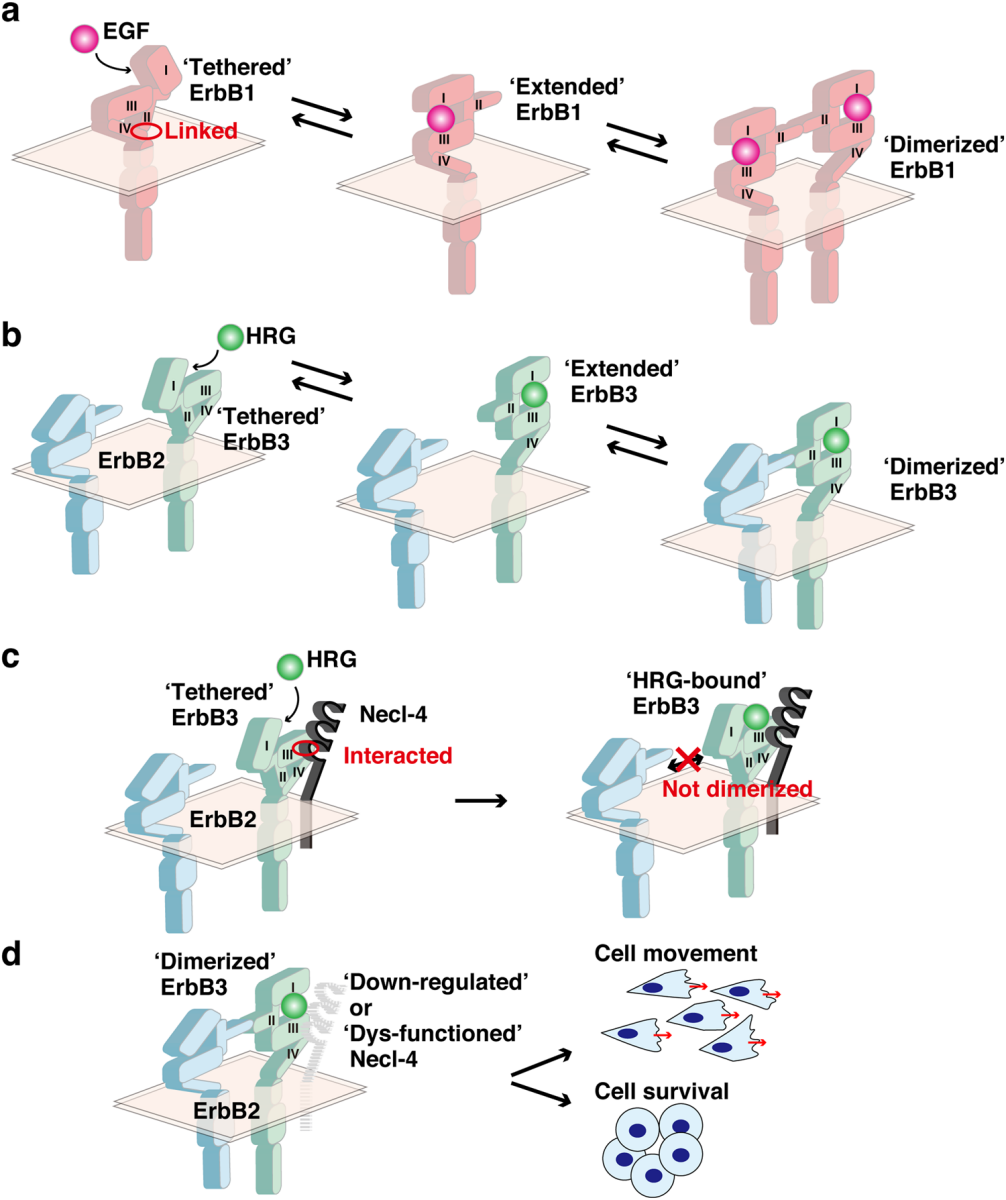
Proposed mechanisms for the ligand-induced activation of ErbB1, -2, and -3 and a model for the inhibitory mechanism of Necl-4 for the HRG-induced activation of the ErbB2-ErbB3 signalling. (**a**) Proposed mechanism for the EGF-induced formation of the ErbB1-ErbB1 homo-dimer. (**b**) Proposed mechanism for the HRG-induced formation of the ErbB2-ErbB3 hetero-dimer. (**c**) A model for the inhibitory mechanism of Necl-4 for the HRG-induced formation of the ErbB2-ErbB3 hetero-dimer. In the absence of HRG, the third Ig-like domain of Necl-4 interacts with the domain 3 of the ‘tethered’ form of ErbB3. This interaction inhibits the HRG-induced formation of the ErbB2-ErbB3 hetero-dimer. (**d**) A model for the activation of the ErbB2-ErbB3 signalling in cancer cells. In cancer cells, Necl-4 is down-regulated or dys-functioned and the inhibitory role of Necl-4 in the HRG-induced formation of the ErbB2-ErbB3 hetero-dimer could be lost and induce cell movement and survival.

Ligands, including hormones, neurotransmitters, and cytokines, bind to specific cell surface receptors, leading to receptor activation and induction of downstream signalling pathway^1, 2^. Ligand-induced dimerization is essential for the activation of single-transmembrane receptors^1^. This mechanism was first shown for the epidermal growth factor (EGF) receptor/ErbB1^3^. One EGF molecule binds to one ErbB1 molecule, leading to the formation of a homo-dimer. Crystallographic studies demonstrate that ErbB1 has an intramolecular ‘tethered’ conformation in the absence of EGF, in which the extracellular domains 2 and 4 are linked^4^ (Fig. 1a). ErbB1 bound to EGF has an ‘extended’ conformation, in which a loop in the domain 2 is released, and serves as a coupling site for interaction with another ErbB1 molecule bound to EGF. This results in the formation of a homo-dimer, which induces the activation of tyrosine kinase in the cytoplasmic region of each receptor, and inter-molecular phosphorylation of tyrosine residues on the other receptor. Phosphorylated tyrosine residues and their flanking short regions interact specifically with Src homology 2 domain-containing molecules and induce the activation of downstream signalling pathways^5^.

There are four subtypes of EGF receptor, ErbB1, -2, -3, and -4, which comprise the ErbB family. The truncated mutant of ErbB1 is an oncogenic protein that forms a homo-dimer without EGF, and transduces cell proliferation signals^6, 7^. ErbB2 also acts as an oncogenic protein and amplification of the *ERBB2* gene is observed in many types of cancer^8^. Gene amplification causes ligand-independent homo-dimerization of ErbB2, which stimulates cancer cell proliferation^9^. ErbB3 overexpression is found in a variety of cancers, and plays a role in cancer cell motility and survival^10^.

ErbB2 does not bind to any ligands known so far but has kinase activity, whereas ErbB3 binds to heregulin (HRG) but lacks kinase activity^10, 11^. Crystallographic studies have revealed that ErbB3 and ErbB1 undergo similar ligand-dependent conformational changes^11^ (Fig. 1b). In contrast, ErbB2 exhibits a different structure because it has an extended conformation in the absence of ligands, with its domain 2 loop available for interaction with ErbB3. In the ErbB2-ErbB3 hetero-dimer formed by the binding of HRG to ErbB3, ErbB2 phosphorylates nine tyrosine residues on ErbB3, leading to the recruitment and activation of downstream signalling molecules, including phosphoinositide 3-kinase, the small G-protein Rac, and the protein kinase Akt^12^. Activation of Rac and Akt promotes cell movement and inhibits apoptosis, respectively.

We previously demonstrated that nectin-like molecule-2 (Necl-2)/cell adhesion molecule 1 *cis*-interacts with ErbB3 via the extracellular region and with the protein tyrosine phosphatase PTPN13, a tumour suppressor, via the cytoplasmic region^13^. Necl-2 inhibits the HRG-induced activation of the ErbB2-ErbB3 hetero-dimer by stimulating dephosphorylation of ErbB3 by PTPN13. Necl-2 is one of the five members of the Necl family, Necl-1, -2, -3, -4, and -5, all of which have one extracellular region with three immunoglobulin (Ig)-like domains, one transmembrane segment, and one cytoplasmic region. Necl-2 acts as a tumour suppressor and is down-regulated by various mechanisms in a variety of cancers^13, 14^.

Necl-4/cell adhesion molecule 4 is another member of the Necl family that has tumour suppressor activity and is down-regulated in several cancer types^15–17^. Our previous research indicated that similar to Necl-2, Necl-4 *cis*-interacts with ErbB3 via the extracellular region and with PTPN13 via the cytoplasmic region, resulting in inhibition of the HRG-induced tyrosine phosphorylation of ErbB3 and Akt^18^. We showed here that Necl-4 *cis*-interacted with ErbB3, irrespective of the presence and absence of HRG, and inhibited the HRG-induced dimerization of ErbB3 with ErbB2 and activation of ErbB3 and its downstream signalling pathways.

## Results

### Ligand-independent interaction of Necl-4 with ErbB3

**Figure 2:**
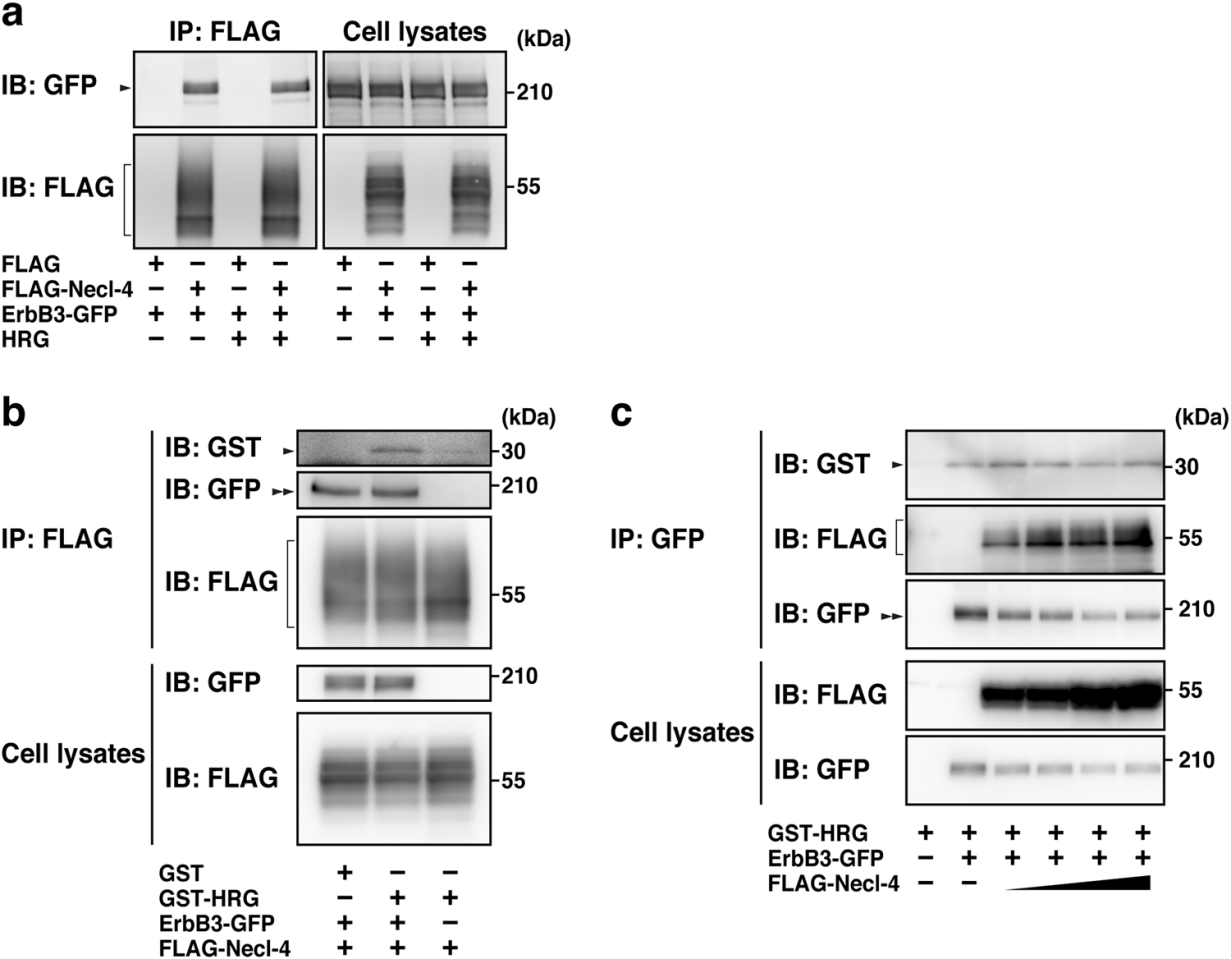
Ligand-independent interaction of Necl-4 with ErbB3. (**a**) Ligand-independent interaction of Necl-4 with ErbB3. HEK293E cells were co-transfected with various combinations of the indicated plasmids and cultured in the presence or absence of GST-HRG. FLAG-Necl-4 was immunoprecipitated using the anti-FLAG mAb, and the samples were subjected to Western blotting using the indicated Abs. Arrowhead, co-immunoprecipitated ErbB3. Square bracket, immunoprecipitated FLAG-Necl-4. (**b**) *In vitro* GST-HRG-binding assay. HEK293E cells were co-transfected with FLAG-Necl-4 and ErbB3-GFP or FLAG-Necl-4 alone as indicated at the bottom. The Necl-4-ErbB3 complex was immobilized on the beads using the anti-FLAG mAb and further incubated with 0.3 μM recombinant GST or GST-HRG at 25 °C for 30 min. The precipitated proteins with the beads were subjected to Western blotting using the indicated Abs. Arrowhead, co-precipitated GST-HRG; square bracket, co-immunoprecipitated Necl-4; double-arrowhead, immunoprecipitated ErbB3. (**c**) No effect of Necl-4 on the GST-HRG binding to ErbB3. HEK293E cells were co-transfected with ErbB3-GFP and different amounts of FLAG-Necl-4 as indicated at the bottom, and the cells were cultured in the presence or absence of 30 nM GST-HRG at 37 °C for 10 min. ErbB3-GFP was immunoprecipitated using the anti-GFP pAb, and the precipitated proteins with the beads were subjected to Western blotting using the indicated Abs. Arrowhead, co-precipitated GST-HRG; square bracket, co-immunoprecipitated FLAG-Necl-4; double-arrowhead, immunoprecipitated ErbB3-GFP. IB, immunoblotting; IP, immunoprecipitation.

We first examined whether Necl-4 interacts with ErbB3 in the absence of HRG. A FLAG-tagged Necl-4 (FLAG-Necl-4) was co-expressed with GFP-tagged ErbB3 (ErbB3-GFP) in HEK293E cells. The cells were starved of serum and cultured in the presence or absence of HRG. When FLAG-Necl-4 was immunoprecipitated using an anti-FLAG monoclonal antibody (mAb), ErbB3-GFP was co-immunoprecipitated with it to a similar extent in the presence and absence of HRG (Fig. 2a). We then examined whether the Necl-4-ErbB3 complex binds to HRG by an *in vitro* binding assay. In this experiment, FLAG-Necl-4 was co-expressed with ErbB3-GFP, and the Necl-4-ErbB3 complex was immobilized on the beads using the anti-FLAG mAb. The beads were further incubated with GST-tagged HRG (GST-HRG) and examined whether GST-HRG binds to the Necl-4-ErbB3 complex on the beads. The binding of GST-HRG to the Necl-4-ErbB3 complex was detected (Fig. 2b), indicating that Necl-4, ErbB3, and HRG form a ternary complex. Since the *in vitro* binding assay alone did not exclude the possibility that Necl-4 inhibits the binding between ErbB3 and HRG, we further investigated whether the presence of Necl-4 affects the binding between ErbB3 and HRG. In this experiment, ErbB3-GFP was co-expressed with FLAG alone or different amounts of FLAG-Necl-4, and the cells were incubated with GST-HRG. When ErbB3-GFP was immunoprecipitated using an anti-GFP polyclonal antibody (pAb) from FLAG-expressing cells, GST-HRG was co-immunoprecipitated (Fig. 2c). Similar levels of GST-HRG were co-immunoprecipitated regardless of the expression level of FLAG-Necl-4 (Fig. 2c). This result excludes the possibility that Necl-4 inhibits the binding between ErbB3 and HRG. Taken together, these results indicate that Necl-4 interacts with ErbB3, irrespective of the presence and absence of HRG.

### Inhibition of the HRG-induced activation of ErbB3 and dimerization of ErbB3 with ErbB2 by the extracellular region of Necl-4

**Figure 3:**
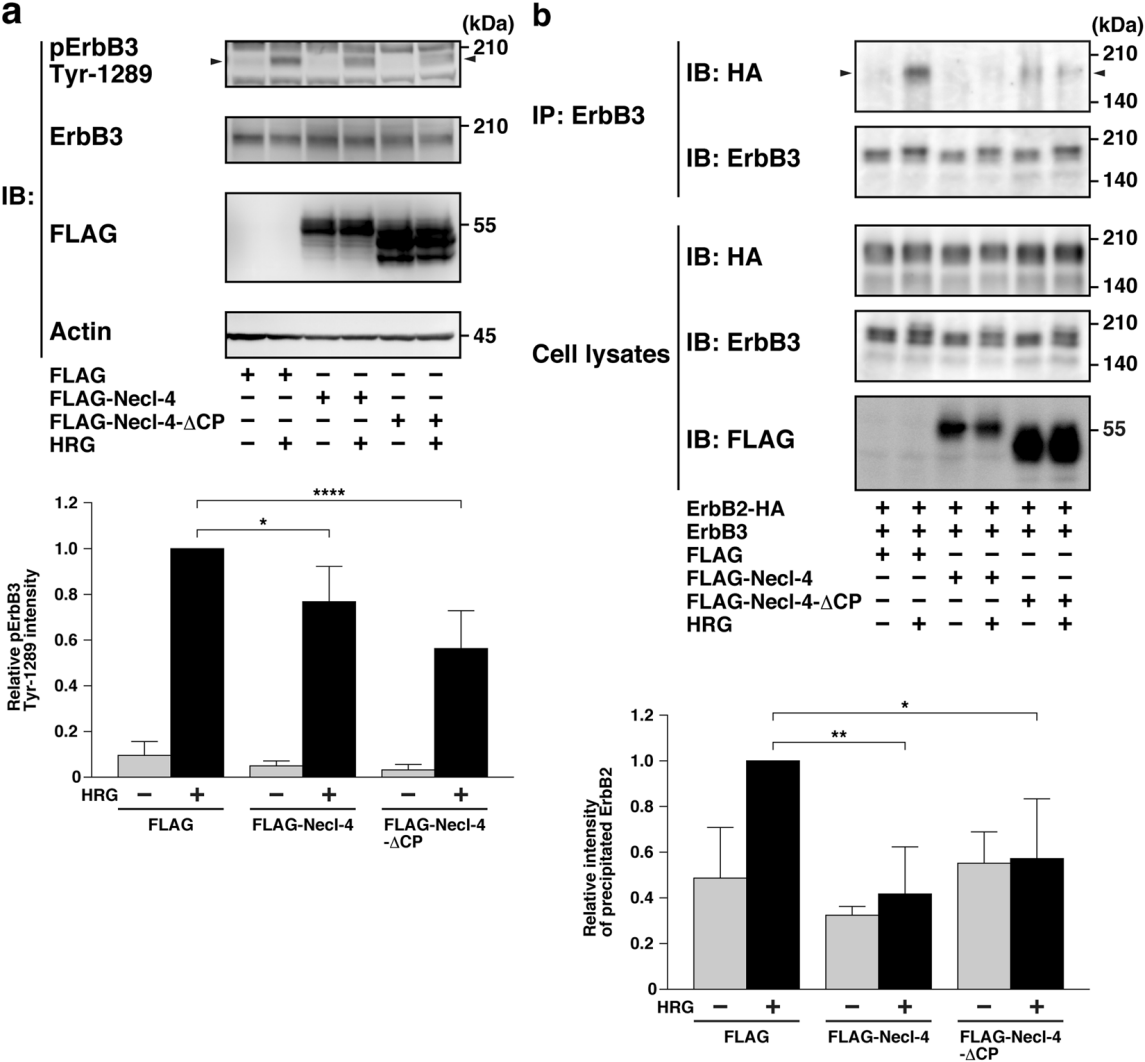
Inhibition of the HRG-induced activation of ErbB3 and dimerization of ErbB3 with ErbB2 by the extracellular region of Necl-4. (**a**) Inhibition of the HRG-induced activation of ErbB3 by the extracellular region of Necl-4. MCF7 cells were transfected with FLAG alone, FLAG-Necl-4, or FLAG-Necl-4-ΔCP. The cells were starved of serum for 24 h and stimulated by 20 ng/ml of HRG for 4 min. The samples were subjected to Western blotting using the indicated Abs. Arrowheads, phospho-ErbB3 on Tyr-1289. The band intensities of the phospho-ErbB3 on Tyr-1289 were normalized to that of the total ErbB3 protein, and the normalized value of the FLAG-expressing MCF7 cells treated with HRG was set as 1.0 (n = 4). *P < 0.05; ****P < 0.0001. (**b**) Inhibition of the HRG-induced ErbB3 dimerization with ErbB2 by the extracellular region of Necl-4. HEK293E cells were co-transfected with various combinations of the indicated plasmids and stimulated by 0.2 μg/ml HRG for 10 min. ErbB3 was immunoprecipitated using the anti-ErbB3 pAb. The samples were subjected to Western blotting using the indicated Abs. Arrowheads, co-immunoprecipitated HA-tagged ErbB2 (ErbB2-HA). The band intensities of ErbB2-HA co-immunoprecipitated with ErbB3 were quantified and the value of the precipitated ErbB2 in the lysates treated with HRG alone was set as 1.0 (n = 3). *P < 0.05; **P < 0.01. IB, immunoblotting; IP, immunoprecipitation.

We next examined whether the HRG-induced ErbB2-mediated activation of ErbB3 is inhibited by the extracellular region of Necl-4. The extracellular region of FLAG-Necl-4 (FLAG-Necl-4-ΔCP), full-length FLAG-Necl-4, or FLAG alone was expressed in human mammary tumour MCF7 cells. These cells were starved of serum and stimulated by HRG. The extracellular region of FLAG-Necl-4 and full-length FLAG-Necl-4, but not FLAG alone, inhibited the HRG-induced phosphorylation of ErbB3 (Fig. 3a). These results suggest that the extracellular region of Necl-4 inhibits the HRG-induced ErbB2-mediated activation of ErbB3.

We then evaluated the effect of Necl-4 on the HRG-induced dimerization of ErbB3 with ErbB2. Previous observations that HRG induces the dimerization of ErbB3 with ErbB2 were first confirmed^19^. HEK293E cells co-expressing ErbB3 and ErbB2 were incubated in the presence or absence of HRG. When ErbB3 was immunoprecipitated using an anti-ErbB3 pAb, ErbB2 was co-immunoprecipitated with it to a small extent in the absence of HRG (Fig. 3b). However, a higher amount of ErbB2 was co-immunoprecipitated with ErbB3 in the presence of HRG than in the absence of HRG, consistent with the previous report^19^. FLAG-Necl-4 was co-expressed with ErbB3 and ErbB2 in HEK293E cells and the cells were incubated in the presence or absence of HRG. When ErbB3 was immunoprecipitated using the anti-ErbB3 pAb, the amount of ErbB2 co-immunoprecipitated with ErbB3 was reduced in the presence of HRG (Fig. 3b). When FLAG-Necl-4-ΔCP was expressed, instead of full length FLAG-Necl-4, the similar inhibitory effect was observed (Fig. 3b). In these experiments, a little constitutive interaction of ErbB3 with ErbB2 was observed in the absence of HRG. The exact reason for this interaction of ErbB3 with ErbB2 in the absence of HRG is not clear, but there might be a weak interaction between ErbB3 and ErbB2 with a high dissociation constant even in the absence of HRG, which is lowered by stimulation with HRG. These results indicate that Necl-4 inhibits the HRG-induced dimerization of ErbB3 with ErbB2.

### Inhibition of the HRG-induced activation of ErbB3 and dimerization of ErbB3 with ErbB2 by the third Ig-like domain of Necl-4

**Figure 4:**
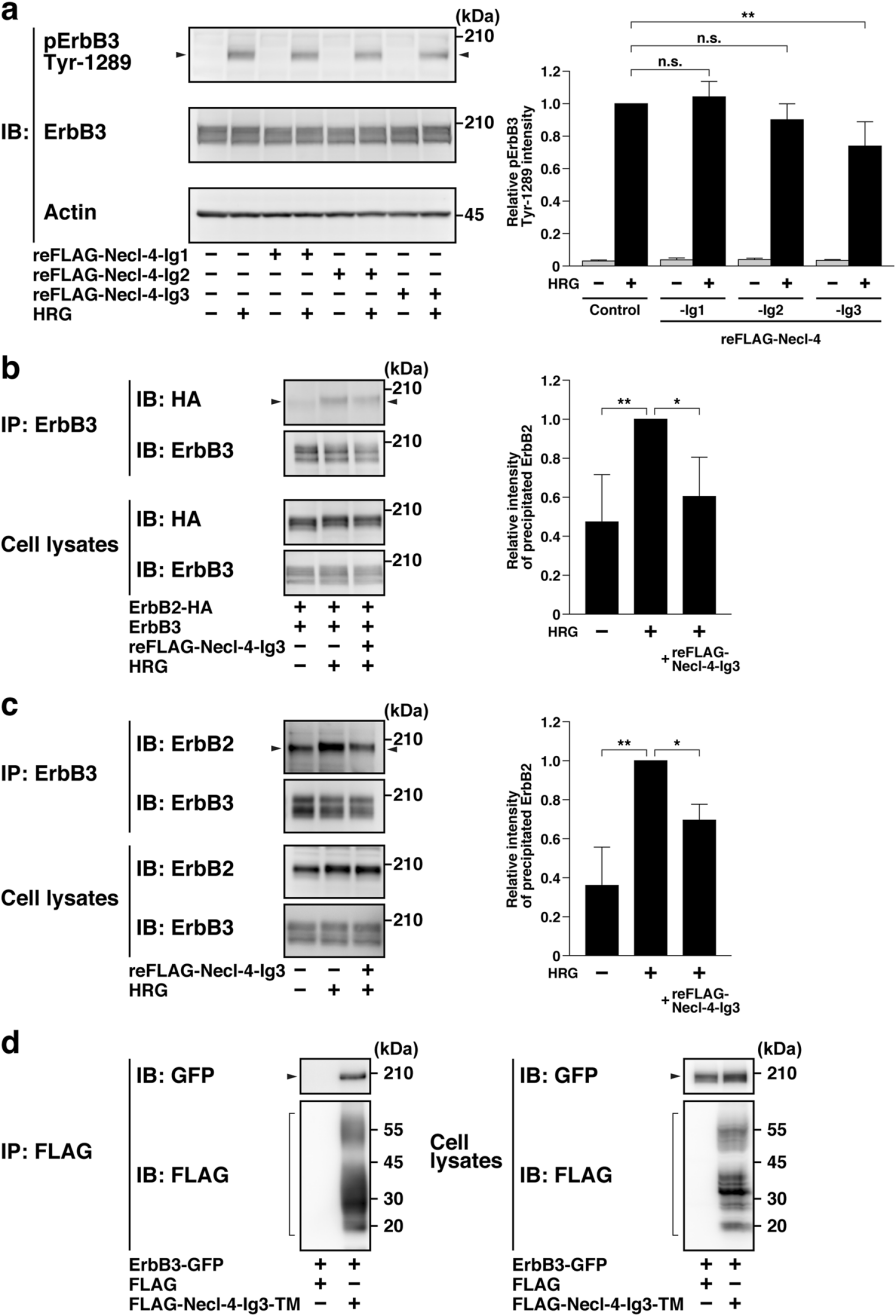
Inhibition of the HRG-induced activation of ErbB3 and dimerization of ErbB3 with ErbB2 by the third Ig-like domain of Necl-4. (**a**) Inhibition of the HRG-induced activation of ErbB3 by reFLAG-Necl-4-Ig3. MCF7 cells were starved of serum and stimulated by HRG for 4 min in the presence of 20 μg/ml of the indicated recombinant Necl-4. The samples were subjected to Western blotting using the indicated Abs. Arrowheads, phospho-ErbB3 on Tyr-1289. The band intensities of the phospho-ErbB3 on Tyr-1289 were normalized to that of the total ErbB3 protein. The normalized value of the MCF7 cells treated with HRG alone was set as 1.0 (n = 3). n.s., not significant; **P < 0.01. (**b**) Inhibition of the HRG-induced dimerization of ErbB3 with ErbB2 by reFLAG-Necl-4-Ig3. HEK293E cells were co-transfected with ErbB3 and ErbB2-HA and treated with 200 μg/ml of reFLAG-Necl-4-Ig3 and stimulated by HRG for 10 min. ErbB3 was immunoprecipitated using the anti-ErbB3 mAb. The samples were subjected to Western blotting using the indicated Abs. Arrowheads, co-immunoprecipitated ErbB2-HA. The band intensities of the co-immunoprecipitated ErbB2-HA with ErbB3 were quantified and the value of the precipitated ErbB2-HA in the lysates treated with HRG alone was set as 1.0 (n = 4). *P < 0.05; **P < 0.01. (**c**) Inhibition of the HRG-induced dimerization of endogenous ErbB3 with ErbB2 by reFLAG-Necl-4-Ig3. MCF7 cells were treated with or without 200 μg/ml of reFLAG-Necl-4-Ig3 and stimulated by HRG for 10 min. Endogenous ErbB3 was immunoprecipitated using the anti-ErbB3 mAb. The samples were subjected to Western blotting using the indicated Abs. Arrowheads, co-immunoprecipitated ErbB2. The band intensities of the co-immunoprecipitated ErbB2 with ErbB3 were quantified and the value of the precipitated ErbB2 in the lysates treated with HRG alone was set as 1.0 (n = 3). *P < 0.05; **P < 0.01. (**d**) Interaction of the third Ig-like domain of Necl-4 with ErbB3. HEK293E cells were co-transfected with various combinations of the indicated plasmids. FLAG-Necl-4-Ig3-TM was immunoprecipitated using the anti-FLAG mAb. The samples were subjected to Western blotting using the indicated Abs. Arrowheads, ErbB3-GFP; square brackets, FLAG-Necl-4-Ig3-TM. IB, immunoblotting; IP, immunoprecipitation.

To determine the domain of Necl-4 required for the interaction with ErbB3, FLAG-tagged recombinant proteins containing the first, the second, or the third Ig-like domain of Necl-4 were prepared from HEK293E cells (reFLAG-Necl-4-Ig1, -Ig2, and -Ig3, respectively). reFLAG-Necl-4-Ig3, but not reFLAG-Necl-4-Ig1 or -Ig2, inhibited the HRG-induced ErbB2-mediated tyrosine phosphorylation of ErbB3 in MCF7 cells (Fig. 4a). reFLAG-Necl-4-Ig3 inhibited the HRG-induced dimerization of ErbB3 with ErbB2 in HEK293E cells (Fig. 4b). Moreover, it inhibited the HRG-induced dimerization of endogenous ErbB3 with endogenous ErbB2 in MCF7 cells (Fig. 4c). The interaction of the third Ig-like domain of Necl-4 with ErbB3 was confirmed by a co-immunoprecipitation assay. The third Ig-like domain of Necl-4 with transmembrane region (FLAG-Necl-4-Ig3-TM) was co-expressed with ErbB3-GFP in HEK293E cells. When the FLAG-Necl-4-Ig3-TM was immunoprecipitated using the anti-FLAG mAb, ErbB3-GFP was co-immunoprecipitated with it (Fig. 4d). These results indicate that the third Ig-like domain of Necl-4 interacts with ErbB3 and is sufficient for inhibiting the HRG-induced dimerization of ErbB3 with ErbB2 and activation of ErbB3.

### Interaction of Necl-4 with the domain 3 of ErbB3

**Figure 5:**
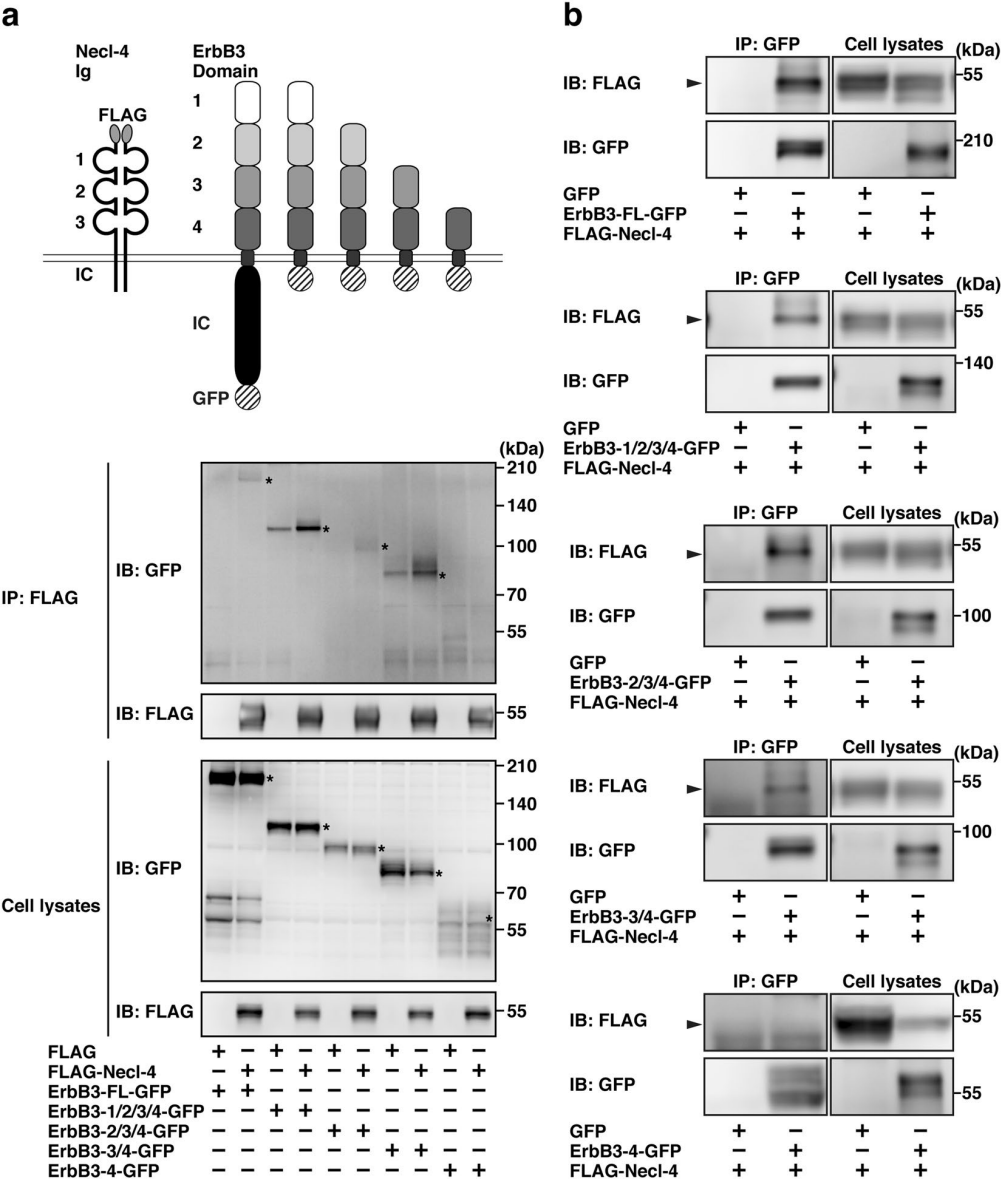
Interaction of Necl-4 with the domain 3 of ErbB3. (**a**) FLAG-Necl-4 immunoprecipitation assay. HEK293E cells were co-transfected with various combinations of the indicated plasmids and cultured in suspension. FLAG-Necl-4 was immunoprecipitated using the anti-FLAG mAb, and the samples were subjected to Western blotting using the indicated Abs. Asterisks, full-length or mutants of ErbB3. (**b**) Reciprocal immunoprecipitation assay. HEK293E cells were co-transfected with various combinations of the indicated plasmids and cultured in suspension. ErbB3-GFP and its mutants were immunoprecipitated using the anti-GFP pAb, and the samples were subjected to Western blotting using the indicated Abs. Arrowheads, full-length or mutants of ErbB3. IB, immunoblotting; IP, immunoprecipitation.

We next attempted to determine the Necl-4-interacting domain of ErbB3 (See Fig. 1b). FLAG-Necl-4 was co-expressed in HEK293E cells with one of the various mutants of the extracellular region of ErbB3, in which the cytoplasmic region, and the domain 1, the domains 1 and 2, or the domains 1, 2, and 3 were deleted, as shown in Fig. 5a. When FLAG-Necl-4 was immunoprecipitated using the anti-FLAG mAb, the ErbB3 mutants, in which the domain 1 or the domains 1 and 2 were deleted, were co-immunoprecipitated with FLAG-Necl-4, but the ErbB3 mutant, in which the domains 1, 2, and 3 were deleted, was hardly co-immunoprecipitated with FLAG-Necl-4 (Fig. 5a). Essentially the same results were obtained in the reciprocal immunoprecipitation assay (Fig. 5b). These results indicate that Necl-4 mainly interacts with the domain 3 of ErbB3. It is concluded from all of these results that the third Ig-like domain of Necl-4 *cis*-interacts with the domain 3 of ErbB3.

### Inhibition of cell movement and anchorage-independent survival of MCF7 cells by the third Ig-like domain of Necl-4

**Figure 6:**
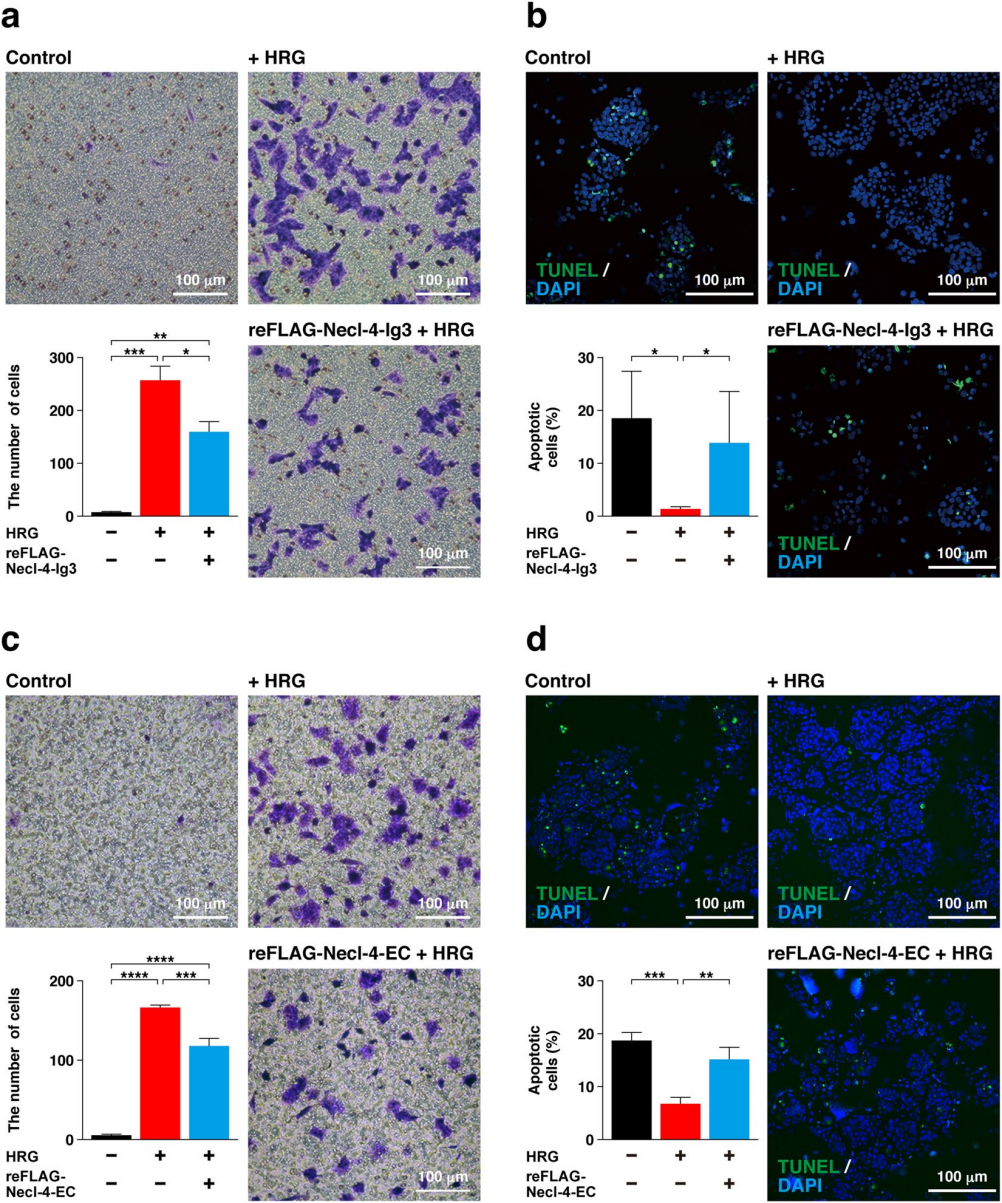
Inhibition of cell movement and anchorage-independent survival of MCF7 cells by the third Ig-like domain of Necl-4. (**a**) Measurement of the activity of the third Ig-like domain of Necl-4 for cell movement using a Boyden chamber assay. MCF7 cells were plated on a cell culture insert (8.0-μm pore membrane filters) in the presence or absence of 10 ng/ml of HRG in the bottom well. Twenty μg/ml of reFLAG-Necl-4-Ig3 was added in both the upper and bottom wells. After incubation at 37 °C for 16 h, the migrated cells were counted by microscopic examination (n = 3). The data are mean ± s.e.m. *P < 0.05; **P < 0.01; ***P < 0.001. (**b**) Measurement of the activity of the third Ig-like domain of Necl-4 for anchorage-independent apoptosis. MCF7 cells were cultured in suspension in the presence of 0.5% fatty acid-free BSA, 1 ng/ml of HRG, and 20 μg/ml of reFLAG-Necl-4-Ig3. After incubation at 37 °C for 48 h, the cells were subjected to a TUNEL assay. The number of apoptotic cells was counted by microscopic examination (n = 3). *P < 0.05. (**c**) Measurement of the activity of the extracellular region for cell movement using a Boyden chamber assay. MCF7 cells were plated on the cell culture insert in the presence or absence of 10 ng/ml of HRG in the bottom well. Fifty μg/ml of reFLAG-Necl-4-EC was added in both the upper and bottom wells. After incubation at 37 °C for 16 h, the migrated cells were counted by microscopic examination (n = 3). The data are mean ± s.e.m. ***P < 0.001; ****P < 0.0001. (**d**) Measurement of the activity of the extracellular region of Necl-4 for anchorage-independent apoptosis. MCF7 cells were cultured in suspension in the presence of 0.5% fatty acid-free BSA, 1 ng/ml of HRG, and 50 μg/ml of reFLAG-Necl-4-EC. After incubation at 37 °C for 48 h, the cells were subjected to a TUNEL assay. The number of apoptotic cells was counted by microscopic examination (n = 3). **P < 0.01; ***P < 0.001.

In the last set of experiments, the effects of the reFLAG-Necl-4-Ig3, a recombinant protein comprising the third Ig-like domain of Necl-4, on HRG-induced cell responses, such as cell migration and anchorage-independent cell survival, were evaluated. reFLAG-Necl-4-Ig3 inhibited both the HRG-induced migration of MCF7 cells, as estimated by the Boyden chamber assay (Fig. 6a), and the HRG-induced anchorage-independent cell survival, as estimated by a TUNEL assay (Fig. 6b). Similar results were obtained by the reFLAG-Necl-4-EC, a recombinant protein comprising the extracellular region of Necl-4, on these cell responses induced by HRG (Fig. 6c and d). These results indicate that the third Ig-like domain of Necl-4 inhibits the HRG-induced signal transduction via the ErbB2-ErbB3 hetero-dimer.

## Discussion

We showed here that Necl-4 *cis*-interacted with ErbB3, irrespective of the presence and absence of HRG, and inhibited the HRG-induced dimerization of ErbB3 with ErbB2 and activation of ErbB3 and its downstream signalling pathways. To the authors’ knowledge, this is the first publication to identify a physiological molecule that inhibits the ligand-induced dimerization of cell surface single-transmembrane receptors. However, several anti-ErbB3 Abs, which inhibit the dimerization of ErbB3 with ErbB2, are under development as anti-cancer drugs^20^. Because Necl-4 inhibits the initial step of the HRG-induced signalling pathway, specifically the dimerization of the cell surface receptor, PTPN13, which interacts with the cytoplasmic region of Necl-4, is not required for the inhibitory action of Necl-4. When Necl-4 does not sufficiently inhibit the dimerization of ErbB3 with ErbB2, PTPN13 may complement the tumour suppressive function of Necl-4.

Crystallographic studies indicate that ErbB3 undergoes ligand-dependent conformational changes led to the proposed mechanism of ligand-induced dimerization of ErbB3 with ErbB2^11^ (Fig. 1b). The interaction between Necl-4 and ErbB3 was induced to a similar extent in the presence and absence of HRG. Thus, in cells expressing ErbB2, ErbB3, and Necl-4, ErbB3 *cis*-interacts with Necl-4 via the extracellular domain 3 of ErbB3 and the third Ig-like domain of Necl-4 to form a hetero-dimer, and this hetero-dimer and ErbB2 are present diffusely on the plasma membrane in the absence of HRG (Fig. 1c, left panel). When cells are stimulated by HRG, HRG binds to the Necl-4-ErbB3 hetero-dimer, but this hetero-dimer fails to interact with ErbB2 to induce the dimerization of ErbB3 with ErbB2 and the activation of ErbB3 (Fig. 1c, right panel). However, the interaction between Necl-4 and ErbB3 was not solely owing to the reduced affinity of ErbB3 for HRG as demonstrated by following results: (1) ErbB3 bound to GST-HRG even in the presence of Necl-4 in the *in vitro* GST-HRG-binding assay as shown in Fig. 2b; (2) the expression of increasing amounts of Necl-4 did not inhibit the binding of GST-HRG to ErbB3 as shown in Fig. 2c; and (3) Necl-4 inhibited the dimerization of ErbB3 with ErbB2, which was induced by a sufficient concentration of HRG as shown in Fig. 3b. However, the detailed mechanism by which the third Ig-like domain of Necl-4 interacts with the domain 3 of ErbB3 to inhibit the ligand-induced dimerization of ErbB3 with ErbB2 remains unclear. One possible mechanism is that Necl-4 prevents the conversion of ErbB3 from a tethered conformation to an extended conformation (Fig. 1c, right panel). Crystallographic studies on the ErbB3-Necl-4 hetero-dimer are needed to address this matter.

We showed here that the third Ig-like domain of Necl-4 was sufficient to interact with ErbB3 and inhibit the HRG-induced dimerization of ErbB3 with ErbB2, but did not clearly describe that the third Ig-like domain of Necl-4 was necessary for inhibiting the HRG-induced dimerization of ErbB3 with ErbB2 and phosphorylation of ErbB3. We previously showed that the extracellular region of Necl-4 is required for its interaction with ErbB3^18^, and we showed here in Fig. 4a that the recombinant protein of the first or second Ig-like domain did not inhibit the HRG-induced phosphorylation of ErbB3 under the condition where the recombinant protein of the third Ig-like domain inhibited this reaction. These results indicate that the first or second Ig-like domain does not interact with ErbB3 at least with efficiency inhibiting the HRG-induced phosphorylation of ErbB3, although we did not examine whether the recombinant protein of the first or second Ig-like domain interacted with ErbB3. We concluded from all of these results that the third Ig-like domain of Necl-4 is necessary and sufficient for its inhibitory effects on the HRG-induced dimerization of ErbB3 with ErbB2 and phosphorylation of ErbB3.

We previously showed that Necl-4 interacts with integrin α_6_β_4_ via its extracellular region^18, 21^, and that integrin α_6_β_4_ interacts with ErbB3^22^. It remains unknown whether the third Ig-like domain of Necl-4 is involved in the interaction with integrin α_6_β_4_ and whether the presence of the third Ig-like domain of Necl-4 affects the interaction between integrin α_6_β_4_ and ErbB3 and its HRG-induced downstream signalling. Further studies are required to verify these possibilities.

It was previously reported that Necl-4 mediates intercellular adhesion through the formation of homo-*trans*-dimers (homophilic *trans*-interaction)^17^, while the activation of the ErbB family members is correlated with their localization at lipid raft^23^. Although we did not examine the possibility that Necl-4 influences the localization of ErbB3, it is possible that Necl-4 takes ErbB3 away from the lipid raft, where the activation of ErbB family members is thought to be induced.

In cancer cells, Necl-4 is down-regulated or dysfunctional and its inhibitory effect on the ligand-induced dimerization of ErbB3 with ErbB2 may be abolished. As a consequence, activation of the hetero-dimer is induced, promoting cancer cell movement and survival (Fig. 1d). The findings of this study may contribute to the development of novel anti-cancer drugs.

## Methods

### Plasmids constructions

The cDNAs for human ErbB2 and ErbB3 were kindly provided from Dr. T. Yamamoto (Okinawa Institute of Science and Technology Graduate University, Japan) and Dr. S. Higashiyama (Ehime University, Japan), respectively. pCAGI-Puro-FLAG-Necl-4 and pFLAG-CMV1-Necl-4-ΔCP were prepared as described^18^. The plasmid encoding FLAG-Necl-4 mutant lacking the signal peptide, Ig1 and Ig2 domains, and the cytoplasmic regions, corresponding to aa 1–25, 26–220, and 351–388, respectively (FLAG-Necl-4-Ig3-TM) were constructed. To obtain various mutants of the extracellular region of ErbB3, in which the cytoplasmic region and the domain 1 (deletion of the signal peptide, the domain 1, and the cytoplasmic region, corresponding to amino acids 1–19, 20–183, and 709–1342, respectively), the domains 1 and 2 (deletion of the signal peptide, the domains 1 and 2, and the cytoplasmic region, corresponding to amino acids 1–19, 20–327, and 709–1342, respectively), or the domains 1, 2, and 3 (deletion of the signal peptide, the domains 1, 2, and 3, and the cytoplasmic region, corresponding to amino acids 1–19, 20–471, and 709–1342, respectively) were deleted, each cDNA fragment was amplified by PCR and inserted into pEGFP-N3 (Clontech, Mountain View, CA). Preprotrypsin signal peptide was inserted into the plasmids of all the ErbB3 mutants at N-terminus. The plasmid encoding the GST-HRG, which was used as GST-NRG1 in the previous study^24^, was kindly provided by Dr. Y. Takada (UC Davis).

### Antibodies and reagents

Mouse anti-actin mAb (clone C4, MAB1501, Merck Millipore, Billerica, MA), horseradish peroxidase (HRP)-conjugated rabbit anti-ErbB2 mAb (clone 29D8, 60388, Cell Signaling Technology, Danvers, MA), rabbit anti-ErbB3 pAb (sc-285, Santa Cruz Biotechnology Inc., Santa Cruz, CA), rabbit anti-ErbB3 mAb (clone D22C5, 12708 S, Cell Signaling Technology), rabbit anti-phospho-ErbB3 (Tyr1289) mAb (clone 21D3, 4791 S, Cell Signaling Technology), mouse anti-FLAG M2 mAb (for immunoprecipitation, F3165, Sigma-Aldrich, St. Louis, MO), rabbit anti-FLAG pAb (for immunoblotting, F7425, Sigma-Aldrich), rabbit anti-GFP pAb (598, MBL International, Nagoya, Japan), mouse anti-HA mAb (MMS-101P, BioLegend, San Diego, CA), and goat anti-GST pAb (27-4577-01, GE Healthcare, Little Chalfont, UK) were purchased from the indicated suppliers. HRP-conjugated secondary Abs for anti-mouse and anti-rabbit IgG, Protein G-Sepharose 4 Fast Flow, and Protein A-Sepharose 4 Fast Flow were purchased from GE Healthcare. HRP-conjugated secondary Abs for anti-goat IgG was purchased from Santa Cruz Biotechnology. HRG-β and fatty acid-free bovine serum albumin (BSA) were purchased from Sigma-Aldrich.

### Protein purification

For the purification of FLAG-tagged proteins containing the first Ig-like domain (amino acids 26–120), the second Ig-like domain (amino acids 121–220), the third Ig-like domain (amino acids 221–323), and the extracellular region (amino acids 26–323) of Necl-4, HEK293E cells were transfected with the indicated plasmids. After the selection of the transfected cells by puromycin, the cells were cultured in the serum-free media (SAFC Biosciences Inc., Lenexa, KS). The secreted Necl-4 Ig proteins were purified from the conditioned media by DDDDK-Ab-conjugated beads (MBL International). The DDDDK-peptide-eluted Necl-4 Ig proteins were ultrafiltered several times with phosphate-buffered saline (PBS) for dialysis and concentration using Amicon Ultra-4 Centrifugal Filter Unit (nominal molecular weight limit 3 kDa, Merck Millipore). GST-HRG fusion protein that includes the EGF-like motif^24^ was expressed in *E*. *coli* using pGEX-2T-HRG-transformed BL21(DE3)pLys Rosetta gami (Novagen, Madison, WI). The *E*. *coli* was cultured in LB medium, and the protein expression was induced by adding 0.2 mM isopropyl β-D-1-thiogalactopyranoside at 25 °C for 2 h. After the induction, the *E*. *coli* was collected and disrupted by BugBuster Master Mix (Novagen). The lysate was clarified by centrifugation at 15,000 rpm for 15 min, and the supernatant was applied into a column of Glutathione-Sepharose 4B (GE Healthcare). The beads were washed by PBS, and further extensively washed by 1% Triton X-114 in PBS to remove endotoxin. To remove the detergent, the beads were washed with PBS again. The GST-HRG was eluted with 5 mM reduced glutathione and immediately concentrated and dialyzed against a dialysis buffer (1 mM reduced glutathione and 2 mM oxidized glutathione in PBS) by ultrafiltration.

### Cell culture and transfection

HEK293E cells were maintained in Dulbecco’s modified Eagle’s medium (DMEM) supplemented with 10% fetal bovine serum, 100 U/ml penicillin, and 100 μg/ml streptomycin, and cultured at 5% CO_2_ at 37 °C. Human breast adenocarcinoma MCF7 cells were maintained in DMEM supplemented with 10% fetal bovine serum, 100 U/ml penicillin, 100 μg/ml streptomycin, and 10 μg/ml insulin, and cultured at 5% CO_2_ at 37 °C. For transient expression of various constructs, Lipofectamine 2000 or Lipofectamine LTX (Thermo Fisher Scientific, Waltham, MA) was used, according to the manufacturer's protocol.

### Assay for the activation of ErbB3

MCF7 cells transfected with various expression vectors were plated at a density of 3 × 10^4^ cells per square centimetre on dishes and cultured for 24 h. The cells were starved of serum with DMEM containing 0.5% fatty acid-free BSA for 24 h and then stimulated by DMEM containing 0.5% fatty acid-free BSA and 20 ng/ml HRG for 4 min. To analyze the effects of the individual Ig-like domain of Necl-4, 20 μg/ml the recombinant protein of FLAG-Necl-4 was also added. The cells were washed with ice-cold PBS and lysed with a lysis buffer (20 mM Tris-HCl at pH 7.5, 1% Nonidet P-40, 10% glycerol, 150 mM NaCl, 1 mM dithiothreitol, 1 mM CaCl_2_, 1 mM MgCl_2_, 10 μM (p-amidinophenyl)methanesulfonyl fluoride, protease inhibitor cocktail (cOmplete, EDTA-free, Roche, Basal, Switzerland), and Phosphatase Inhibitor Cocktail 2 and 3 (Sigma-Aldrich)). The lysates were then heated at 80 °C in a sodium dodecyl sulfate (SDS) sample buffer (67 mM Tris-HCl at pH 6.8, 2% SDS, 100 mM dithiothreitol, 5% sucrose, and 0.005% bromophenol blue) for 2 min and subjected to SDS-polyacrylamide gel electrophoresis (SDS-PAGE), followed by Western blotting.

### Western blotting

The samples separated on SDS-PAGE were transferred to poly(vinylidene difuloride) membranes (Merck Millipore). After being blocked with 5% skim milk in Tris-buffered saline plus 0.05% Tween 20, the membranes were incubated with the indicated Abs. After being washed with Tris-buffered saline plus 0.05% Tween 20 three times, the membranes were incubated with HRP-conjugated anti-rabbit, anti-mouse, or anti-goat IgG Ab. The signals for the proteins were detected using Immobilon Western Chemiluminescent HRP Substrate (Merck Millipore).

### Co-immunoprecipitation assay

HEK293E cells were co-transfected with various combinations of the indicated plasmids and cultured for 48 h. MCF7 cells were treated with or without 200 μg/ml of reFLAG-Necl-4-Ig3 and stimulated by 0.2 µg/ml HRG for 10 min. The cells were washed with PBS and lysed with the lysis buffer. The lysates were rotated for 30 min, subjected to centrifugation at 12,000 × g for 15 min, and incubated with the anti-FLAG mAb, the anti-ErbB3 pAb, or the anti-ErbB3 mAb-conjugated Protein A or Proteins G Sepharose beads at 4 °C for 2 h. After the beads were extensively washed with the lysis buffer, bound proteins were eluted by heating at 80 °C in the SDS sample buffer for 2 min, and subjected to SDS-PAGE, followed by Western blotting.

### Boyden chamber assay

Polycarbonate membrane cell culture inserts (8.0-μm pores, Corning Inc., Corning, NY) were coated with serum for 1 h at 4 °C. MCF7 cells were suspended in DMEM containing 0.5% fatty acid-free BSA and seeded at a density of 5 × 10^4^ cells per insert in the presence or absence of 10 ng/ml of HRG in the bottom well. Twenty μg/ml of reFLAG-Necl-4-Ig3 or 50 µg/ml of reFLAG-Necl-4-EC was added in both the upper and bottom wells. After incubation at 37 °C for 16 h, the inserts were washed with PBS. The cells were fixed using 4% paraformaldehyde in PBS and stained with crystal violet. The cells that had not migrated were removed by wiping the top of the membrane with a cotton swab. The number of migrated cells in five randomly chosen fields per filter was counted by microscopic examination. The images were acquired using Olympus CKX41 inverted microscopy with a LCAchN 20 ×/0.40 numerical aperture objective lens and a SP-601UZ digital camera (Olympus Corporation, Tokyo, Japan) at room temperature.

### Anoikis assay

To prevent cell adhesion, plates were coated with a solution of poly(2-hydroxyethyl methacrylate) (Sigma-Aldrich) dissolved at 20 mg/ml in ethanol. To coat plates, 100 μl of poly(2-hydroxyethyl methacrylate) solution was added to each plate per square centimetre, and the plates were kept at room temperature for 2 days until the solvent had completely evaporated. MCF7 cells were cultured in suspension using a poly(2-hydroxyethyl methacrylate)-coated dish in the presence of 0.5% fatty acid-free BSA, 1 ng/ml of HRG, and 20 μg/ml of reFLAG-Necl-4-Ig3 or 50 µg/ml of reFLAG-Necl-4-EC. After incubation at 37 °C for 48 h, the cells were collected and attached to Matsunami adhesive silane-coated slide glass (Matsunami Glass, Osaka, Japan) using a Cytospin 4 Cytocentrifuge (Thermo Scientific), and apoptotic cells were detected by TUNEL assay using the DeadEnd Fluorometric TUNEL System (Promega, Madison, WI) according to the manufacturer’s protocol. DNA was visualized with DAPI, and apoptotic cells were detected by fluorescein-12-dUTP. The samples were analysed by fluorescence microscopic examination. The images were acquired using Nikon C2 confocal system (Nikon, Inc., Tokyo, Japan) with a Plan Apo 20 ×/0.75 numerical aperture objective lens (Nikon, Inc.) at room temperature under the control of NIS-Elements AR Analysis software 4.20 64-bit (Nikon, Inc.). The images were processed using Photoshop CS6 (Adobe, San Jose, CA).

### Statistical analysis

The data are reported as means ± s.d. unless otherwise noted. Statistical significance was analysed using one-way ANOVA with post-hoc Dunnett’s multiple-comparison test or Tukey’s multiple-comparison test for comparison between more than two groups. Prism software (GraphPad Software Inc., La Jolla, CA) was used for analysis of statistical significance. A *P*-value less than 0.05 was considered statistically significant.
